# Von Hippel-Lindau Disease: A Rare Radiological Case Report of a Symptomatic Patient and His Asymptomatic Genetic Counterpart

**DOI:** 10.7759/cureus.12925

**Published:** 2021-01-26

**Authors:** Sachin Khanduri, Nazia Khan, Saif Malik, Shivangi Katara, Mariyam Fatima

**Affiliations:** 1 Radiodiagnosis, Era's Lucknow Medical College and Hospital, Lucknow, IND

**Keywords:** von hippel-lindau disease (vhl), renal cell carcinoma (rcc), pheochromocytomas, pancreas

## Abstract

Von Hippel-Lindau (VHL) disease is an inherited syndrome manifested as a benign and malignant tumor. It is an autosomal dominant syndrome diagnosed approximately in 1 in 36,000 people.

We report a case where male siblings presented with the involvement of bilateral kidneys and the multi-cystic lesion on the pancreas in both. Reverse transcription polymerase chain reaction (RT-PCR) was conducted to detect the VHL gene, which turned out to be a significant finding in our study. The rare involvement of both pancreas and kidneys was noted in the siblings with VHL in the present study.

In patients with VHL-associated tumour presentations, the most frequent detection of pathogenic variants in the VHL gene is the result of directed genetic testing or inherited cancer gene panels. The presence of renal and pancreatic involvement is rare but a significant finding present within the family member who needs to be screened.

## Introduction

Von Hippel-Lindau (VHL) syndrome is an inherited syndrome manifested as a benign and malignant tumor. It is an autosomal dominant syndrome diagnosed approximately in 1 in 36,000 people [1-2]. The spectrum of VHL associated tumors includes clear cell renal cell carcinomas (RCCs), pheochromocytomas, serous cystadenomas and neuroendocrine tumors of the pancreas, hemangioblastoma of cerebellum and spine, retinal capillary hemangioblastoma, and the endolymphatic sac tumors of the middle ear.

## Case presentation

**Figure 1:**
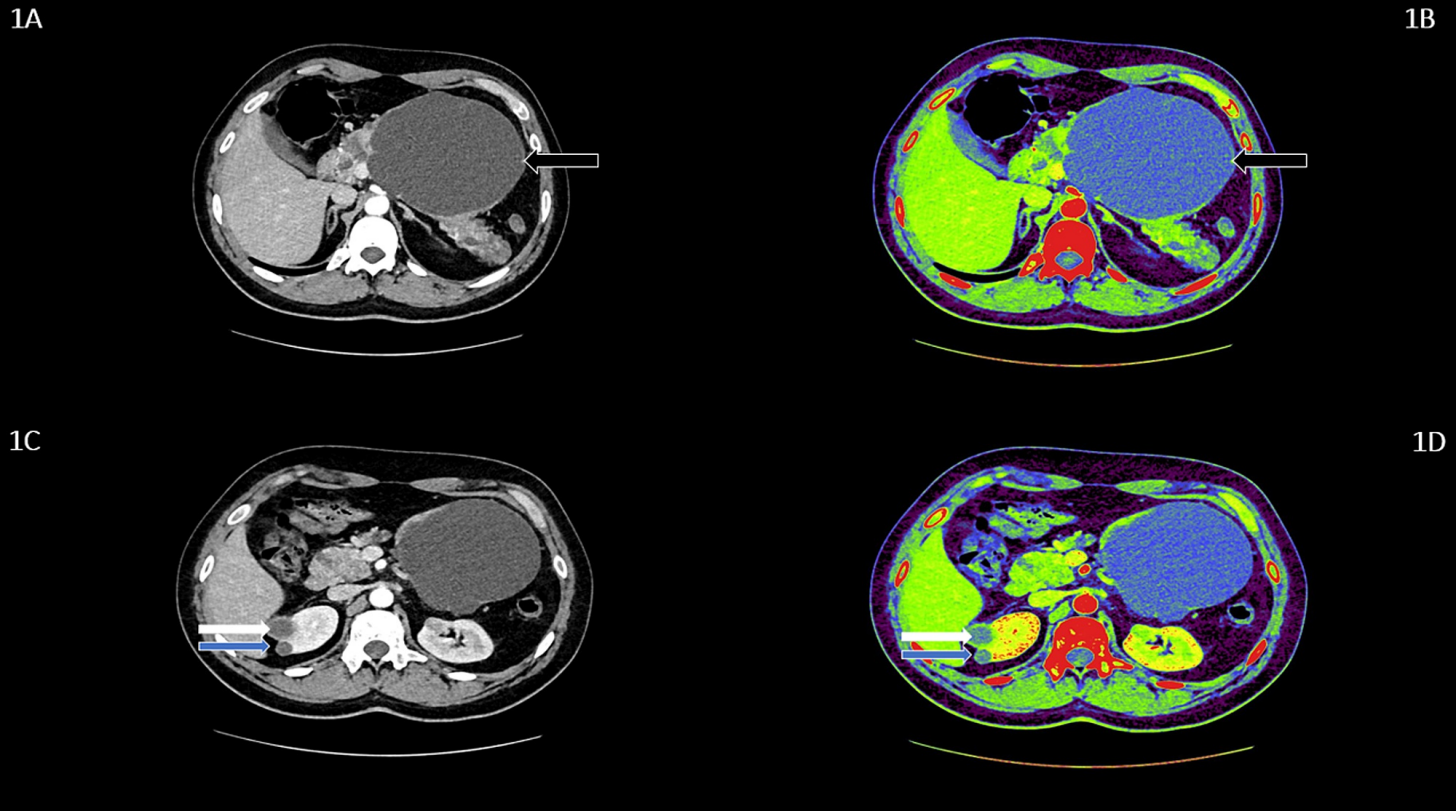
CECT whole abdomen of the symptomatic patient demonstrating pancreatic and renal manifestations 1A and 1B: Axial CECT abdomen shows a pancreatic cystic lesion with few septations, showing the focal calcification in the body of the pancreas (black arrows). 1C and 1D: Axial CECT abdomen shows a well-defined cystic lesion, showing the peripheral enhancement and a peripherally placed enhancing soft tissue attenuation nodule within, at the upper pole of the right kidney, suggesting a neoplastic etiology (white arrows); and a hypodense cystic lesion at the upper pole of the right kidney, suggesting renal cyst (blue arrows). CECT: contrast-enhanced computed tomography

**Figure 2:**
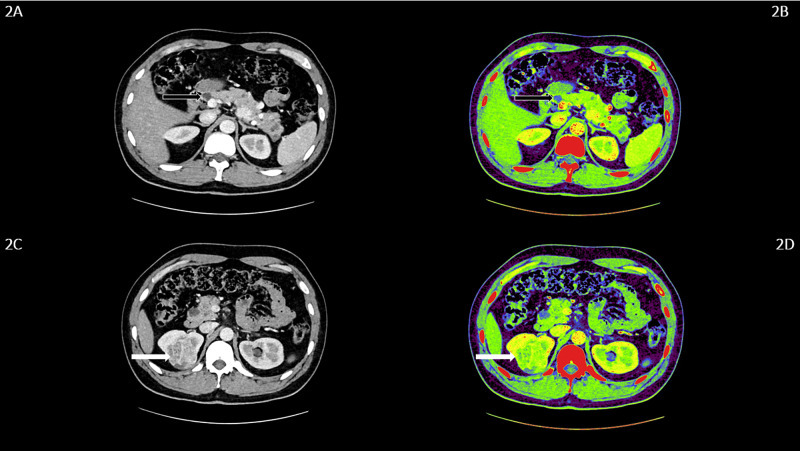
CECT whole abdomen of the asymptomatic patient demonstrating pancreatic and renal manifestations 2A and 2B: Axial CECT abdomen shows multiple small, simple cysts involving the entire pancreatic parenchyma (black arrows). 2C and 2D: Axial CECT abdomen shows a well-defined heterogeneously enhancing mass lesion, with few cystic areas within, arising from the upper pole of the right kidney, suggesting a neoplastic etiology (white arrows). CECT: contrast-enhanced computed tomography

**Figure 3:**
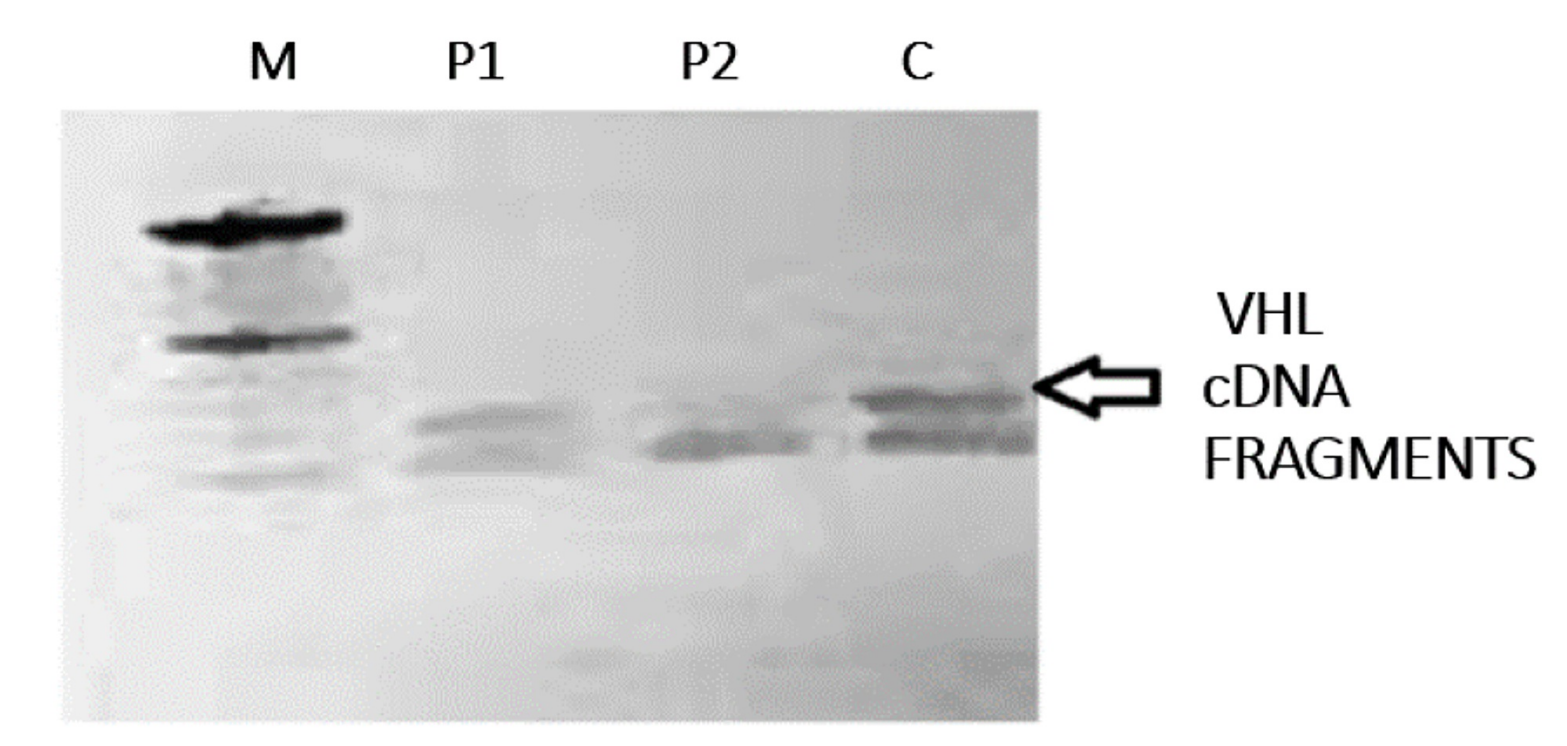
RT-PCR for VHL cDNA fragment testing VHL cDNA fragments (arrow) amplified by RT-PCR. Lanes - M: molecular weight marker; P1: symptomatic patient; P2: asymptomatic patient; C: pooled normal controls. The lower band in each sample are primer dimers, N=2. VHL: von Hippel-Lindau disease; cDNA: complementary deoxyribonucleic acid; RT-PCR: reverse transcription polymerase chain reaction

A 32-year-old male presented with a history of fever and abdominal pain particularly over the epigastric region. On examination, a palpable lump was appreciated in the epigastric region. On further examination, it was found that subtle hematuria was present on urine analysis, and the presence of hypertension was detected on physical examination. The patient was subjected to further radiological evaluations. The abdominal ultrasonography (USG) and CT were performed. They showed a significant disease extensive with a multitude of pancreatic cystic lesions. The largest measured 12.6x11.8x11.7 cm noted with few septations showing the focal calcification in the body of pancreas (Figure 1A, 1B). There were multiple hypodense cystic lesions, the largest measuring 5.0x4.4 mm was noted at the upper pole of the right kidney. A similar finding was noted on the middle pole of the left kidney, with the largest measuring 6.3x5.1 mm. A well-defined cystic lesion showing the peripheral enhancement and a peripherally placed enhancing soft tissue attenuation nodule within, at the upper pole of the right kidney suggested neoplastic etiology (Figure 1C, 1D). The sibling of the patient was also subjected to investigation suspecting VHL. He was asymptomatic at the time of the investigation. The findings on the CT abdomen of the sibling, who was a 34-year-old male, revealed similar findings. There was presence of multiple small cystic areas with simple cysts involving the entire pancreatic parenchyma (Figure 2A, 2B). A well-defined heterogeneously enhancing mass lesion measuring 4.4x4.2 cm with a few cystic areas within was noted arising from the upper pole of the right kidney, which suggested neoplastic etiology (Figure 2C, 2D). The left kidney showed hyperdense calculus measuring 6.4x4.5 mm in the proximal ureter. A hypodense water density cyst measuring 7.6x6.9 mm was noted in the middle pole of the left kidney. The reverse transcription polymerase chain reaction (RT-PCR) for VHL complementary deoxyribonucleic acid (cDNA) fragment was performed, which showed a significant finding in our patients (Figure 3).

## Discussion

VHL gene is mapped to chromosome 3p25 and was cloned in the year 1990s, with further research into the knowledge about the gene function extending over the next twenty years. The VHL gene products function as a tumor suppressor protein [3]. In order to produce VHL-associated tumours, there must be a lack of expression of the second normal allele either by somatic mutation, or by the deletion of the second allele, or by the hypermethylation of the promoter. In sporadic renal cell cancer, the inactivation of VHL by somatic mutation of both alleles is very common.

Data conflict with the link of particular germline mutations and the occurrence of renal cell carcinoma. For example, according to the analysis, the total risk of renal cell carcinoma in 138 families with VHL disease was comparable in those with significant deletions and mutations in the VHL gene relative to those with missense mutations [4]. On the other hand, in another study of 274 individuals in 126 unrelated families, gene defects resulting in truncated or missing protein or large rearrangements resulted in an increased occurrence of renal cell carcinoma relative to missense mutations (81% versus 63%) [5].

In patients with VHL-associated tumour presentations, the most frequent detection of pathogenic variants in the VHL gene is the result of directed genetic testing or inherited cancer gene panels. The American College of Medical Genetics and Genomics (ACMG) publishes guidelines for disclosing incidental findings (now referred to as secondary findings) while conducting complete exome or genome sequencing [6-7].

Families with VHL have been divided into type 1 and 2 based on the likelihood of developing pheochromocytoma. Type 1 disease has a lower risk of developing pheochromocytomas (type 1A) and also a lower risk of pheochromocytomas and RCC (type 1B). Whereas type 2 disease has a higher risk for developing pheochromocytoma. The type 2A and 2B families have a low and higher risk of RCC, respectively, in them. However, type 2C are characterised by the development of pheochromocytomas only with no evidence of RCC or hemangioblastoma in them [8]. Pancreatic abnormalities are common in patients with VHL disease. In a multi-institutional study of 158 consecutive patients from 94 affected families, 77% had lesions in the pancreas, including cysts (70%), serous cystadenomas (9%), and neuroendocrine tumors (9%) [6,7]. Easy pancreatic cysts and serous cystadenomas can be asymptomatic even though the radiological appearance is dramatic. However, such lesions can cause epigastric pain and discomfort [9-10]. Pancreatitis and pancreatic disease are exceptionally rare disorders, although some degree of exocrine pancreatic dysfunction has been reported.

Surveillance protocols focus on hemangioblastomas (including retinal capillary hemangioblastomas), RCCs, pheochromocytomas, and audiology, given the increased risk of endolymphatic sac tumors (ELST) in patients with VHL. Surveillance recommendations may need to be adapted to the individual patient, taking into account the patient’s current or prior tumor diagnoses. However, all individuals with VHL, even if currently asymptomatic, should understand that they may develop manifestations of VHL disease and will benefit from following surveillance guidelines. Several organisations have revised surveillance protocols for contemporary imaging and laboratory diagnostic approaches. The International Committee of Clinicians Caring for Children with VHL was assembled in 2016 as part of the American Association for Cancer Research (AACR) Childhood Cancer Predisposition Workshop. The panel reviewed both the American and European VHL regimens and issued surveillance guidelines, including enhanced strength and early initiation (Table 1) [11].

**Table 1:** Surveillance for individuals with a pathogenic or likely pathogenic variant in VHL. VHL: Von Hippel-Lindau disease

Surveillance	Age to initiate	Interval	Comments
Dilated retinal examination	Before 1 year	Every 6 to 12 months	Annually after age 30
History and physical examination by physician informed about VHL	1 year	Every year	
Blood pressure and pulse	2 years	Every year	
Metanephrines	5 years	Every year	Plasma free metanephrines preferred but 24-hour urine fractionated metanephrines can be used.
MRI of the brain and spine	11 years	Every 2 years	Performed with and without contrast (without contrast if pregnant). Can be coordinated with MRI of the abdomen. Include thin cuts through the posterior fossa and petrous temple bone. One-time MRI of the internal auditory canal at age 15 years.
Audiology	11 years	Every 2 years	
MRI of the abdomen	15 years	Every 2 years	Performed with and without contrast (without contrast if pregnant). Assess kidneys, pancreas, and adrenal glands. Can be coordinated with MRI of the neuroaxis.

In this case, however, the diagnosis of VHL was done by contrast-enhanced computed tomography and was subsequently confirmed by RT-PCR amplification.

## Conclusions

VHL continues for the entire course of life. Patients should be screened for tumours and cysts that develop at different sites along with suitable intervention whenever required. Patients with VHL suffer from trouble caused by multiple tumours and cysts involving various organs, most commonly, brain, kidney, and pancreas. A bright prospect for this disease is the innovation of molecular targeting anti-angiogenic drugs in the future. Patients must be cared for by well-trained specialists throughout their lives to improve the prognosis caused by the above-mentioned conditions.
